# Implementation of fixed-dose combination therapy for secondary prevention of atherosclerotic cardiovascular disease among Syrian refugees in Lebanon: a qualitative evaluation

**DOI:** 10.1186/s12913-022-08040-z

**Published:** 2022-06-04

**Authors:** Adrianna Murphy, Ruth Willis, Éimhín Ansbro, Sahar Masri, Nour Kabbara, Tonia Dabbousy, Sola Bahous, Lucas Molfino, Pablo Perel, Philippa Boulle

**Affiliations:** 1 Department of Health Services Research and Policy, London School of Hygiene and Tropical Medicine (LSHTM), London, UK; 2 Médecins Sans Frontières, Operational Centre Geneva (MSF-OCG), Beirut, Lebanon; 3 Department of Internal Medicine, Lebanese American University, Beirut, Lebanon; 4 MSF OCG, Geneva, Switzerland; 5 Department of Non-communicable Disease Epidemiology, LSHTM, London, UK

## Abstract

**Background:**

We report findings of a qualitative evaluation of fixed-dose combination therapy for patients with established atherosclerotic cardiovascular disease (ASCVD) attending Médecins Sans Frontières (MSF) clinics in Lebanon. Cardiovascular disease is a leading cause of death and disability worldwide, and humanitarian actors are increasingly faced with the challenge of providing care for chronic diseases such as ASCVD in settings where health systems are disrupted. Secondary prevention strategies, involving 3–5 medications, are known to be effective for patients at risk of heart attack or stroke, but supply and adherence are challenging in humanitarian settings. Fixed dose combination therapy, combining two or more medications in one tablet, may be a strategy to address this.

**Methods:**

The evaluation was nested within a prospective mixed-methods study in which eligible ASCVD patients were followed for 1 year during (i) 6 months of usual care then (ii) 6 months of fixed dose combination (FDC) therapy. After 1 year, we conducted in-depth interviews with a purposive sample of patients, MSF staff and external stakeholders. Interviews focused on acceptability and sustainability of the fixed dose therapy intervention. Interview data were analysed thematically, informed by thea Theoretical Framework of Acceptability. Additional attention was paid to non-typical cases in order to test and strengthen analysis.

**Results:**

Patients and health care providers were positive about the FDC intervention. For patients, acceptability was related to ease of treatment and trust in MSF staff, while, for staff, it was related to perceived improvements in adherence, having a good understanding of the medication and its use, and fitting well with their priorities for patient’s wellbeing. External stakeholders were less familiar with FDC therapy. While external clinicals expressed concerns about treatment inflexibility, non-clinician stakeholder interviews suggested that cost-effectiveness would have a major influence on FDC therapy acceptability. Sustainability was tied to the future role of MSF care provision and coherence with the local health system.

**Conclusions:**

For patients and clinic staff, FDC was an acceptable treatment approach for secondary prevention of ASCVD disease in two MSF clinics in Lebanon. Sustainability is more complex and calls for better alignment of care with public systems.

**Supplementary Information:**

The online version contains supplementary material available at 10.1186/s12913-022-08040-z.

## Background

Cardiovascular disease (CVD), including ischemic heart disease and stroke, is the leading cause of death and disability worldwide. Each year, CVD causes almost 18 million deaths and 30 to 50 million non-fatal CVD events. Four of five CVD deaths occur in low-and middle-income countries (LMICs), and half are among people under 70 years of age, many in the prime of their productive years [1]. In contexts affected by humanitarian crises – caused by conflicts, natural disasters, or food insecurity - CVD is a growing public health problem. As crises have become more protracted and more often occur in middle income countries, where non-communicable diseases in general are more prevalent, [2, 3] humanitarian actors are increasingly faced with the challenge of providing long-term care for chronic diseases such as CVD [4]. They are faced with this challenge in contexts where the existing health systems are often poorly coordinated, fragmented and under-resourced, and capacity to ensure continuity of care when humanitarian actors leave is unclear.

The risk of death or morbidity from CVD can be reduced with cost-effective pharmacological treatment. Optimal treatment for secondary prevention of heart attack and stroke among those with established CVD requires 3–4 daily pills including blood-pressure lowering, cholesterol-lowering and antiplatelet drugs. However, only approximately 48% of people with CVD in high-income countries are on optimal treatment, and in LMICs this number is less than 5% [5]. Although there are no data specifically from humanitarian crisis settings, the number of eligible people on optimal treatment in these settings may also be low, due to inconsistent access to health facilities and medicines. One potential strategy to address low rates of treatment for secondary prevention of CVD is the use of fixed-dose combination therapy (FDCs). FDCs for CVD combine two or more blood-pressure lowering medications and a statin, with or without aspirin, and can improve adherence, reduce blood pressure, cholesterol and fatal and non-fatal CVD events, and can be cost-effective [6–8].

The argument for use of FDCs for secondary prevention of CVD is increasingly clear, particularly in settings with limited healthcare resources. Combination therapies have been instrumental in the scale-up of HIV, tuberculosis and malaria care in settings with weak health infrastructure, scarce resources and high patient loads [9]. They offer potential advantages for treatment of CVD too, including simplified procurement, distribution, storage and prescription, and improved adherence by patients. Despite these potential advantages, the use of FDCs for CVD globally, including in humanitarian crisis settings, is low [7]. Improving access to FDCs in humanitarian crisis settings requires better evidence on the factors affecting implementation of FDCs in these contexts, to inform effective intervention and advocacy strategies [10].

The study described in this paper is part of a larger mixed methods study aimed to evaluate the implementation of an FDC treatment strategy (including an ACE inhibitor, statin and aspirin) for patients with established atherosclerotic CVD (ASCVD; history of coronary heart disease, ischaemic cerebrovascular disease, or peripheral artery disease) in two Médecins Sans Frontières (MSF) clinics in Lebanon. Separate reports will present the results of the quantitative study, which found that the FDC treatment strategy improved adherence, blood pressure and cholesterol control, and of the costing study. We report here the results of a qualitative evaluation of the acceptability and sustainability of the intervention.

### Study setting: the MSF clinics for Syrian refugees in Abdeh and Dar Al Zahara (DAZ), Lebanon

As of the end of 2020, Lebanon hosted 879,529 registered Syrian refugees, although the total number of Syrian refugees in the country is estimated to be closer to 1.5 million [11]. Syrian refugees are spread throughout Lebanon, mainly in community rather than camp-based settings. Among adult Syrian refugees, the prevalence of CVD is estimated to be 3.3% [12]. The health system in Lebanon is pluralistic, comprising a mix of private, public faith-based and international and national NGO providers, but is dominated by the private sector and oriented towards curative care. More than 80% of the Ministry of Public Health budget is spent on private hospitals and pharmaceuticals and only 5% is spent on primary health care [13]. Since early 2012, MSF has been providing free, integrated primary care for Syrian refugees and vulnerable Lebanese in Akkar and Tripoli governorates in North Lebanon. Two MSF-supported clinics, Dar Al Zahara (DAZ) in Tripoli and Abdeh in Akkar, have offered integrated NCD care since 2012 and 2015, respectively. In 2017, a cross-sectional study of 514 patients in Abdeh and DAZ found that 83.2% of the patients were prescribed three recommended drugs: at least one blood pressure lowering drug, statin and antiplatelet but that self-reported adherence was 54.35% in DAZ and 82.04% in Abdeh. The most common self-reported reasons for poor adherence among patients were not understanding the prescription and the decision not to take certain drugs either for the whole week or on a certain number of days [14].

For this FDC implementation study, patients were eligible for enrolment if they were aged 18 years and older, were attending either DAZ or Abdeh clinics, had ASCVD and were receiving (or were eligible to receive) a multiple pill treatment regimen for secondary prevention (aspirin, statin, BP lowering medication). Patients were enrolled on a rolling basis as they attended for routine appointments during a four-month period from February to May 2019. Usual care (treatment of established ASCVD with aspirin, a statin and at least one blood pressure lowering drug) continued for 6 months after the last patient was enrolled (June–November 2019). After the 6-month period of usual care, an FDC was introduced into routine care to replace the multiple tablet combinations of ASCVD secondary prevention drugs, for all eligible clinic patients enrolled in the study (December 2019–May 2020). Thus, all eligible ASCVD patients without contraindications and who agreed were switched from usual care to Trinomia® (Ferrer) (atorvastatin 20 mg (a statin), ramipril 2.5, 5 or 10 mg (an ACE-inhibitor), and aspirin 100 mg); Trinomia is the most widely approved FDC for secondary prevention (approved in over 20 countries) and was approved by the Lebanese Ministry of Public Health in November 2016. Out of 521 eligible patients, 460 were retained at their 6-month consultation. Of these, 418 (90.9%) were switched to Trinomia and 42 (9.1%) remained on usual care. Reasons for non-switching included: contra-indication (*n* = 23, 5.0%), patient preference (*n* = 11, 2.4%), hyperkalaemia (*n* = 4, 0.9%), gastrointestinal symptoms (*n* = 1, 0.2%), dizziness (*n* = 1, 0.2%), and other reasons (*n* = 2, 0.4%). Patients did not need to already be taking ACE-inhibitors to be enrolled in the study (i.e. they may have been on other blood pressure-lowering medication classes). All patients’ blood pressure medications were reviewed. Patients who were eligible for the study but were not already on an ACE-inhibitor, and where an ACE-inhibitor was clinically appropriate, were put on an ACE-inhibitor prior to the switch to Trinomia. No patients enrolled in the study had their statin dose changed by the switch to Trinomia. Eligible patients were taking either 40 mg of Simvastatin or the equivalent 20 mg of the more potent Atorvastatin prior to the switch to Trinomia. Patients were informed about the components and doses of Trinomia through an information leaflet and discussion with their physician. Patients switched to Trinomia continued to take their other prescribed treatments as usual. A concise treatment protocol and specific training sessions and materials were developed to support doctors in initiating, maintaining, and adjusting (if necessary) the FDC and other concomitant drugs. The 460 patients retained at their 6 month consultation were followed for a further 6 months, during which 351 (84.0%) of the 418 patients who had switched remained on the FDC, 34 (8.1%) discontinued it and 33 (7.9%) (*n* = 33) were lost to follow-up. In September 2019, after study enrolment had begun, a decision was taken by MSF to slowly wind down operations at DAZ and Abdeh clinics and transfer patients to local clinics registered with the Lebanese Ministry of Public Health. Patients participating in the implementation study remained registered at DAZ and Abdeh clinics until completion of the study and were then transferred to local clinics.

The primary objective of this study is to qualitatively evaluate the implementation of the FDC strategy, focusing specifically on perceptions of its acceptability and sustainability among patients, MSF staff and external stakeholders.

## Methods

### Data collection

We conducted in-depth semi-structured interviews with ASCVD patients, MSF staff and external stakeholders. To be eligible for qualitative interviews, patients had to have participated in the quantitative component of the study. Invited patients were selected purposively to represent a range of demographic categories (gender, age group, educational level) that is broadly representative of the clinic population of ASCVD patients, but randomly from within those categories. We also purposively over-sampled patients who declined to switch to Trinomia or who stopped taking it during the course of the implementation study. The final sample size for patients was determined by the stage at which data saturation was reached (i.e. where a sufficient number of interviews were conducted to capture key themes and the addition of more interviews was not generating new themes). MSF staff were purposively selected to ensure inclusion of one member from each staff role involved in delivering ASCVD care in the clinics. External stakeholders were purposively selected from institutions or organisations with a stake in public health care delivery for ASCVD in Lebanon but external to MSF. Interviews were conducted between July 2020 – March 2021.

Interviews focused on the acceptability and sustainability of the FDC intervention but were open-ended to allow for the participant to guide the discussion as much as possible. The topic guides for patients, staff and external stakeholders are included as Additional file 1. Each topic guide was reviewed by the project team, piloted and adjusted as needed for coherence and flow. To address acceptability, we drew on the Theoretical Framework of Acceptability developed by Sekhon et al. [15] This framework consists of seven constructs that define acceptability, outlined in Table 1.

**Table 1:** Theoretical Framework of Acceptability [15]

Construct	Meaning
Affective attitude	How an individual feels about the intervention
Burden	The perceived amount of effort that is required to participate in the intervention
Ethicality	The extent to which the intervention has good fit with an individual’s value system
Intervention coherence	The extent to which the participant understands the intervention and how it works
Opportunity costs	The extent to which benefits, profits, or values must be given up to engage in the intervention
Perceived effectiveness	The extent to which the intervention is perceived to be likely to achieve its purpose
Self-efficacy	The participant’s confidence that they can perform the behaviour(s) required to participate in the intervention

The assessment of sustainability is a developing field, with various proposed frameworks from different disciplines [16]. For the purposes of our study, we used the definition of sustainability proposed by Gruen et al., which addresses a programme’s or intervention’s ‘capability of being maintained at a certain rate or level.’ [17] We focused on factors that might challenge or support the internal (within MSF clinics) and external (within the Lebanese health system) sustainability of the FDC intervention.

Eligible participants were approached by telephone (patients, some staff), email (stakeholders, some staff) or face-to-face (some staff), provided with an information sheet and invited to sign the informed consent form in order to be included in the study. Additional file 2 gives details of recruitment approach, data collection mode and setting for each participant group. No invited participants refused to participate in the qualitative study or dropped out during the study. Interviews were conducted by four researchers (3 female; 1 male) with experience of conducting qualitative interviews, led by a senior qualitative researcher (AM, PhD) who provided study specific training and feedback. Interviews with patients were conducted by the study research assistant (NK) and research manager (SM), trained native Arabic speakers, in Arabic. Interviews with staff were conducted by trained external researchers (AM, RM) in their preferred language (English or Arabic) and with external stakeholders in English. Interview duration ranged from 30 to 60 minutes. All interviews were audio recorded, transcribed and where necessary, translated into English.

### Data analysis

Transcriptions were analysed in NVivo12 Plus©, using a thematic approach, reflexively coding data for emerging themes [18]. Analysis involved initial open coding of data, identification of core codes and constant comparative analysis to develop categories and generate themes. Emergent themes related to acceptability were compared against the Theoretical Framework on Acceptability and where possible categorised according to the Framework’s constructs. Findings were scrutinized by a second team member to enhance analytic credibility.

## Results

We interviewed 32 patients, 11 MSF staff involved in the coordination and delivery of NCD care and 8 external stakeholders. Details of patient demographics and staff roles are included in Table 2.

**Table 2:** Qualitative interview participants

Participant group	Number (%, for patient group)
*Patients*	*32 (100%)*
Male	22 (69%)
Secondary education or above (vs Primary or none)	9 (28%)
Non-switcher or discontinued	7 (21%)
*MSF Staff (Roles included: general practitioner, clinic manager, NCD nurse, NCD referent doctor, mission pharmacist, project pharmacist, medical co-ordinator)*	*11*

The main themes generated by the interviews with each participant group are outlined in Table 3, organised by the construct that they address.

**Table 3:** Themes on acceptability and sustainability of FDC intervention, by participant group

Construct	Theme		
	*Patients*	*MSF staff*	*External stakeholders*
**ACCEPTABILITY**			
Affective attitude	Makes life easier *Among those who did not switch or discontinued:* Fear of unfamiliar Lack of control	Treatment improvement	Perception of high risk Ok for the poor/uninsured
Intervention coherence	Attribution of side effects	Clarity regarding pill and how it works	
Burden	Easier than previous treatment	Early effort, long-term reward	
Self-efficacy	High capability to execute	Empowered by information	
Ethicality	Helps achieve patient goals	Fit with values/patient welfare	
Opportunity costs			Sacrifices treatment flexibility
Perceived effectiveness	Make patients feel better	High efficiency Improved efficiency	
Other	Trust in (MSF) doctors		
**SUSTAINABILITY**			
Challenges	Dependence on MSF/lack of faith in external system Financial barriers	Inconsistent supply of drugs to clinic Lack of coherence Lack of transition plan	Background context (i.e. political, social, health system factors outside of MSF control) Changing established practice of clinicians Lack of commercial interest outside MSF
Supporting factors		Time investment Contextualisation of intervention within local health system and political circumstances. Intervention as advocacy	MSF as catalyst/precedent for change Stakeholder engagement Integration into health system Price/economic crisis as opportunity

### Patients - acceptability

Overall patients had positive experiences of the polypill. This seemed driven by the perception of one pill being “easier” than three or more separate pills and something they felt capable of adhering to. As one patient pointed out, “it’s one pill so doesn’t let you forget to take it”. It was also driven by the perception of the drug as an effective medicine that fit well with patients’ priorities or goals, namely feeling well and not experiencing side effects. For example, when asked if the polypill fulfilled those criteria that are important to them in a medicine, one patient responded:

*“100%. I’ll tell you why. Because, I’m feeling very well with it, I’m not complaining at all from any side effects or pain or anything like that at all. I’m very comfortable with it. If I weren’t comfortable, I would have stopped it. I would have come here and told them to remove it.”* [Patient 10]

One prominent theme that explained FDC acceptability among patients that did not fit well with any constructs from the Theoretical Framework on Acceptability is a strong trust in doctors, and specifically MSF doctors, and what they prescribe. This trust seemed to be as important for acceptability as (or perhaps even linked to) how patients feel about the drug itself, and seemed to even override some patients’ attribution of any unpleasant side effects to the FDC. The following two examples from patient interviews demonstrate this trust:

*“I don’t know anything at all. I will take whatever you give me.”* [Patient 5]

*“You know, you explained to us from the beginning … and if it weren’t good, you wouldn’t have explained it or even given it to us.”* [Patient 19]

Among the small group of non-switchers, (i.e. those patients who declined to switch to Trinomia, [*n* = 11, 2.4%] in the overall study), there appeared to be fear or reluctance related to an unfamiliar drug. They felt secure about the medications they were currently on and didn’t want to introduce any risk of side effects from a new drug. In one example, this fear was related to a previous experience of hair loss that a patient had attributed to a blood pressure medication he had been prescribed. In response to being asked whether he was told Trinomia consisted of the same three drugs he was already on, he replied:

*Patient: (yes), they told me … I didn’t test the medication, but they told me it has 3 types in 1 pill … But with this experience I didn’t dare. The blood pressure medication I know it, the diabetes medication I know it, the blood thinner I know it and the lipid [ … ]*

*Interviewer: so you trust the medication you are currently taking and you wouldn’t change them?*

*Patient: yes I don’t want to change them* [Patient 15]

And among those who discontinued Trinomia (*n* = 34, [8.1%] in the overall study), there seemed to be a similar attribution of side effects to Trinomia and a preference for a drug regimen with which they were familiar and that they felt “worked better” for them.

*Patient: I started to worry about having to swallow this pill today. It became a burden (laughs) … because once I swallow it, it means 2-3 hours later I would get these disturbances in my body … I wouldn’t be normal … What do I want with this medication, I don’t want it anymore …*

*Interviewer: do you think that these symptoms are caused by something specific in the medication or it might be because of something else?*

*Patient: no, it might because it’s one mass*

*Interviewer: you mean one combined pill?*

*Patient: yes, this is my belief. Because originally … there wasn’t one pill I swallowed, except the blood thinner and another one, the regulator, I swallowed them together. Other than that, there were hours between one pill and another.*

*Interviewer: when the doctor here prescribed again the blood thinner, blood pressure and lipid medication separately, did these symptoms go away or did they persist?*

*Patient: not at all, I went back to normal, as if nothing’s wrong.* [Patient 11]

Other non-switchers described FDCs as something that resulted in a lack of control. They raised concerns that FDCs did not allow them the flexibility to adjust their blood pressure medication in response to fluctuations in their blood pressure readings, which they were monitoring regularly themselves.

*“What I’m afraid about this combined medication, is that … one doesn’t know how the blood pressure is going to be like … If it’s low and you take the pills altogether like this... it will be difficult” [Patient 32]*

### Patients - sustainability

With respect to sustainability, it was rare that patients focused on the theoretical sustainability of the FDC intervention (i.e. would they continue taking it forever if they could). The greater focus was on the real-life unsustainability of the intervention in light of the fact that MSF was closing its clinics and patients were being referred to local primary health clinics where FDC would not be supplied. Linked to the trust in MSF doctors was a theme that presented a challenge to sustainability, the apparent lower level of trust of affordable health care providers outside of MSF described by some patients. As one patient explained:

*“I’ll tell you something. If a doctor from outside, tells me there this combined medication, I would never take it, because honestly there is no trust at all. But in Doctors without Borders [MSF], I trust them, so I accepted the idea. That’s why as an experience, I accepted it. But be sure, that if it was from outside, even if it was a doctor, I would have refused.”* [Patient 10]

Also emerging as a challenge to sustainability of the intervention was the dependence on MSF for health care and for medicines, especially financially.

*“Now if you tell me MSF will stop providing me with the medication, I will stop taking the medication, I don’t have money at all to buy it, honestly. My wife and I are living in a (warehouse). There is only god. If MSF stops giving the medication, I will have to stop.”* [Patient 4]

### MSF staff - acceptability

Staff were similarly positive overall about the polypill and felt it was an improvement in treatment and that they understood the pill well and how it works. The view that the treatment was an effective improvement over a multi-pill regime seemed due to perceived improvements in adherence. Acceptability among staff also appeared to be supported by the fact that the FDC fit with staff’s values, in that it supported improving patient welfare: *“We’re seeing the best way to provide this medication and the reason for providing it is to avoid the patient making a mistake, and that’s a huge thing.”* [Staff Member 3].

The overall reduction in work burden also played an important role in acceptability. Staff seemed to view the FDC intervention as something that required early investment of effort, but that over time paid off with a lighter workload. One nurse described the following experience when asked about if and how their work changed:

*“Of course, after we started with Trinomia, things changed a bit … I had to focus on this medication, provide proper education to the patient … After that, even in the simplest things, like writing on the file, rather than writing simvastatin, and so on, you just put Trinomia … It saves our time, saves the patient’s time … . all these things helped honestly.”* [Staff Member 6]

What also emerged as crucial to acceptability was a theme related to self-efficacy, the sense of empowerment that staff felt due to the training and support provided to them about the FDC. This highlighted how important it was to hold early training sessions with staff that would allow them to ask questions and become comfortable with the new drug and with prescribing it.

*“Honestly the first training … we were like the patients, we didn’t know anything about it … we had a lot of questions: “What would the patient ask me?” … “How would I answer?” … “What if the patient refused?” … they told us everything about it and that it’s approved by the authorities and the Ministry of Health, so it was reassuring that I’m not giving the patient a medication where he’d go to the neighbours and they’d ask him “what are you taking?” or something … so in a way I’m protected, legally speaking. And when we knew everything about it, it was okay, we didn’t feel there was a problem anymore … we were convinced … we should be convinced as well to be able to convince the patient.”*[Staff Member 5]

Hesitation regarding the acceptability of FDC treatment among staff appeared to be related not to the pill itself but to the ethicality of introducing a new treatment that could not be guaranteed once MSF closed its clinics (a theme that arises again when considering sustainability of FDCs). Staff expressed a feeling of discomfort with introducing a switch to patients’ treatment regime only to have to likely switch it back once discharged from MSF. As one staff member described:

*“The only thing I’d say I didn’t like about this is, first, it’s still a newly introduced drug. So, the problem is when I want to introduce a drug to a patient, I want to make sure it’s sustainable. In case I will no longer be available to give it to him for free, the study stops … I don’t know how accessible this is for people with low income or no income like the refugees. So yeah mainly this is the compromise. Yes, it’s a nice combination and its very convenient but how accessible is it? Is this going to affect the patient? Can he get it later? Can he afford it later? These are the things that I think contradict what I believe in.”* [Staff Member 8]

To alleviate their concerns around ethicality and to accept FDCs as in line with their values, staff seemed to make a conscious decision to view the FDC intervention as part of a larger effort to advocate for use of FDCs for Syrian refugees and Lebanese more generally. Several staff discussed framing the study and the introduction of FDCs as working together with patients to generate evidence that would support the use of FDCs as a standard treatment option, and thus lead to longer-term treatment improvement.

*“ … there was a clear need to clarify our position, to explain the rationale of the decision (to introduce FDCs despite imminent MSF departure) … … Finally we got something in the middle … we explain to the people that we saw, that we keep seeing the added value to push for a fixed dose combination feasibility study in a context like Lebanon … we saw that maybe our patients will not be benefitting in the long term, or (it’s) not a very sustainable decision, but that (it) will contribute to general evidence to keep advocating for a change … You know, so basically, we frame in that sense, and after that, we … we shift a little bit this approach and we take … we create accountability … we start to treat the patient as a partner.”* [Staff Member 9]

### MSF staff - sustainability

The prospect of internal sustainability (i.e. could an FDC intervention be sustainable within MSF clinics in Lebanon generally), seemed to hinge on whether appropriate time could be invested in training staff and supply of the drug to the clinic was maintained (likely related to ruptures in supply of previously used drugs, as Trinomia’s supply was consistent from the beginning to end of study). As long as this was possible it was viewed that FDCs were a treatment improvement that would have equal applicability and benefit in other MSF clinics. More complex themes emerged related to the external sustainability of FDCs (i.e. whether it could be sustained by the local health system outside of MSF), and are linked to the issues of ethicality raised above. The key challenge to sustainability of FDCs appeared to be a perceived lack of coherence of the intervention with the local health system, and the unlikelihood that MSF practice would simply be adapted by that system.

*“In general, I think it (FDC) is easy and effective but you need everyone to be committed to it … we’re not staying forever.. MSF is going to close … so is it something other doctors would follow? I feel there are many difficulties and challenges because every doctor works with a specific company that markets medication … It will also be very difficult for MoPH to impose it … so I feel there are a lot of difficulties for later on … For our work (at MSF), it’s easy, but maybe for later on, it’s difficult.”* [Staff Member 5]

### External stakeholders - acceptability

The acceptability of FDCs for secondary prevention of ASCVD for external stakeholders depended on their role. Most non-clinicians were unfamiliar with FDCs before this study and thus did not have strong views on this treatment approach, but highlighted that in theory, a treatment that improved adherence would be acceptable as long as it was cost-effective. External stakeholder interviews with practicing clinicians highlighted a common theme raised in other studies of clinicians’ views of FDCs for CVD – that they limit treatment flexibility, come with risks, and are inappropriate for specialised treatment (a view that may reflect some misunderstanding of the dosage options currently available with FDCs). As one participant explained:

*“I was telling you that we rather keep our … some kind of freedom to tailor the treatment because statins in cardiac patients are important and we have to reach a very low level of LDL cholesterol, which is not very easy to handle with a fixed dose, because we may start with 10 mg, 20, but we might end up with 40 mg and add another treatment on top of the statin. For this reason, we are a little bit … not at ease to use the fixed dose, the polypill [ … .] … I think this (public dispensaries) is the first place to use it … in these kinds of facilities … the second is the GPs, and the third is the specialists, maybe at the end because certainly the scope of its use there (in specialist facilities) will be very very poor.”* [Stakeholder 4]

### External stakeholders - sustainability

With respect to sustainability, external stakeholders seemed uncertain that the FDC intervention could be sustained, and this uncertainty seemed driven by the view of MSF as operating outside the health system.

*“MSF do a very good job, but it’s outside this logic … I mean, they have their clinics which are not very sustainable in the long run because they will not be integrated in the national system … Because you know, for example, the clinic of MSF, they create new clinics...and these clinics are not belonging to this country.”* [Stakeholder 1]

On the other hand, a key emergent theme that could support sustainability is that of MSF’s opportunity to act as a precedent for innovative treatment approaches, including FDCs. This seemed to be an opportunity that could only be exploited if MSF engaged stakeholders at an early stage and throughout the process, especially clinicians whose “hearts and minds” would have to be changed, and formally integrated interventions as much as possible into the existing health system’s circumstances. The below quotations demonstrate this theme.

*“Then it’s good to organize a meeting with the Ministry of Public Health, invite donors, and stakeholders and then to do follow up. Not just to organize one meeting and it’s forgotten. Advocacy is something that is in the long run … say okay, we pilot, I don’t know in two clinics … this is the result, we invite you to pilot this. Then go to the donors and say okay why don’t you pilot this in another. And then organize a joint meeting with the Ministry of Public Health and a joint meeting with the UN and WHO, why not, to discuss technicalities. Because if you don’t push push push, nothing will move forward.”*[Stakeholder 1]

*“It needs to be clear who will manufacture the product and how you will integrate it within the existing supply chain of the country, who should basically be on board when you get their buy in … the Ministry of Public Health, their technical department, the Primary Healthcare Department, definitely the Secretary General, also embedded within the guidelines of the Ministry of Health … then how … there’s any governance issue related for instance to the standard operating procedures and the clinical guidelines because there is a chronic disease guideline, for example for the diagnosis, treatment, what would be the dosage and so on, so there is this layer of how it will fit into that.”*[Stakeholder 3]

Stakeholders also seemed to perceive the financial crisis in Lebanon as a “window of opportunity” in which to advocate for FDCs, given their potential for being cost-saving, but that advocacy would still be required to support their use.

*“Particularly in these days with the financial crisis, I think that it’s not really hard to convince the cardiologist of the necessity to adopt such a drug. But of course they will argue, they like to argue.”* [Stakeholder 6]

## Discussion

Our study is the first to qualitatively evaluate the implementation of FDCs for ASCVD in a humanitarian crisis setting. We identified key themes that may explain the acceptability and sustainability of FDCs for secondary prevention of ASCVD among refugees in Lebanon, as perceived by patients, health workers and external stakeholders. While our primary goal was to generate practical insight that could support MSF in implementing FDCs in their NCD programmes, our findings can inform the implementation of FDCs and other similar treatments for chronic diseases in humanitarian crisis settings.

Many of the themes generated by our interviews align with the constructs outlined in the Theoretical Framework on Acceptability and with findings from other studies of FDCs in other settings. In particular, several randomised controlled trials and surveys that have investigated FDC acceptability among patients and health workers have reported favourable views of FDCs for high-risk patients, driven mostly by the ease, convenience and simplicity of the pill, and related improvements in adherence [19–28]. Also, like previous studies, we identified concerns among prescribers about what they viewed as inflexibility of FDCs in dosing, but notably this was not expressed by any MSF staff included in our study, all of whom underwent training on FDCs. This inflexibility was also raised as an issue by patients. Together, these findings highlight the importance of communicating the benefits of increased simplicity of FDCs to both patients and health workers, but also strengthening information and advocacy campaigns targeting health workers specifically, to highlight the potential for dose modification with FDCs. With Trinomia, for example, formulations include different doses of ramipril (an ACE inhibitor) and statin (although the latter was not available in Lebanon at the time of the study), enabling easy titration to achieve blood pressure and lipid control, whilst maintaining a formulation of one combination tablet. In Lebanon such campaigns might be channelled through professional societies (for example the Society of Cardiologists), but the best approaches to support education on FDCs for prescribers will likely be context-specific.

Our study also expands on our understanding of the role that perceived treatment ease or simplicity plays in FDC acceptability among health workers. It does so by highlighting also the importance of ensuring health workers feel sufficiently informed and empowered to enact their role in prescribing FDCs. This finding is consistent with previous evaluations of the implementation of healthcare interventions in general, and specifically with research that has led to and has since been informed by Normalisation Process Theory (NPT). NPT explains the factors that act as barriers or facilitators of routine incorporation of healthcare innovations into practice. It describes processes undertaken by intervention actors to make sense of, engage with, enact and reflect on new ways of working. Within these processes, the actors’ understanding of their role in enacting the intervention, their confidence in doing so, and the sense that they are the “right people” to do so are all important to supporting implementation [29, 30]. NPT also highlights the process by which actors internalise the values or benefits of an intervention. Our study shows how sensitive this process can be to specific contextual factors – while MSF staff all agreed on the clinical benefits of FDCs, their acceptance of the intervention was challenged by concerns regarding its short term and uncertain nature. These concerns were alleviated through a process of collectively making sense of the intervention as a partnership with patients to generate evidence that might improve treatment options for ASCVD patients in the long term.

With respect to sustainability, our findings from all study groups – patients, MSF staff and external stakeholders – centre around the fragmentation of care between humanitarian NGOs like MSF and governmental health care providers. The role of humanitarian actors in ensuring sustainability of interventions is a general issue of debate in the humanitarian research and practice community [31, 32] and is of particular relevance when considering care for chronic diseases. Previous work, including our own, has highlighted challenges to sustainability relating to dependence on NGO expertise and uncertainty of what would be offered after their departure [33, 34]. The findings of this study add further support to the argument that with chronic disease programmes in particular, meaningful engagement on the part of NGOs with the health system is important to ensure sustainability [33]. Such engagement may be particularly challenging in a context like Lebanon, where there is a plurality of health care providers, including a large private sector and public facilities run by religious groups, national and international NGOs. This plurality combined with minimal expenditure on primary care compared to secondary or tertiary care creates challenges for adopting public health approaches [31]. While the specific ways to support successful adoption of such approaches would vary by context, our study identified approaches that are likely generalisable, including engaging key stakeholders in the intervention from an early stage, to facilitate alignment with local health system policies and resources, developing advocacy and educational initiatives supported by evidence of intervention cost-effectiveness, especially targeting prescribers in the case of new drug treatments. Experiences of MSF and others in the treatment of HIV have demonstrated the success of similar approaches [35]. Specific approaches have involved establishing formal partnerships with local government and other health authorities, governed by memoranda of understanding; integrating the programme with other health services, including with drug supply chains; and advocating for scale-up and incorporation of successful programme elements into national guidelines, as was recommended with community ART distribution programmes for HIV [36, 37]. An internal evaluation of advocacy activities related to HIV treatment in Mozambique highlighted the importance of systematically mapping the interests of all actors in the health systems and the resources available to them, and having trained staff within the NGO that are dedicated to advocacy activities and approach these with humility.

### Limitations

Our findings should be interpreted in light of some limitations. First, we only included patients who were attending MSF clinics and enrolled in the FDC study. It is possible that these participants are not representative of the larger population of Syrian refugees with ASCVD in Lebanon. It is also possible that patients and staff responded to our questions with what they imagined the interviewer wanted to hear (so called “desirability bias”). To mitigate this risk, we emphasised that interviewers were not involved in designing the FDC intervention, and participants appeared to respond openly in interviews, including with criticisms of the intervention. Our study findings were also likely affected by the unexpected closure of MSF operations in both clinics. While we were able to collect data prior to clinic closures, our interviews were inevitably affected by the planned closures, limiting our ability to fully explore sustainability of the intervention within MSF operations. The themes on wider sustainability, particularly those raised by external stakeholders, may be reflective of the Lebanese context specifically, and any initiatives to implement and sustain chronic disease interventions in other settings must be contextualised. However, as discussed, our findings are consistent with previous work on implementation of NCD interventions in humanitarian settings and can contribute to a growing general understanding of the key factors required to support sustainability in these contexts.

## Conclusions

FDCs for secondary prevention of ASCVD, implemented in two MSF clinics in Lebanon, appear to be an acceptable treatment approach to patients and MSF staff. Acceptability is supported by the simplicity of the drug, patient trust in MSF doctors, and empowerment of staff with training and information about FDCs. Sustainability appears more complex – within MSF clinics, an FDC intervention is perceived as sustainable as long as supply and support of staff is consistent. However, external sustainability of the FDC, as with any innovation in chronic care by humanitarian organisations, is challenged by the nature of humanitarian health care, namely the fragmentation and lack of coherence between NGO and governmental health provision. Taking advantage of opportunities to better align care will require resources and early and consistent commitment to stakeholder engagement and advocacy.

## Supplementary Information

**Additional file 1.**

**Additional file 2.**

## Data Availability

The minimal data set underlying the findings of this paper are available on request, in accordance with the legal framework set forth by Médecins Sans Frontières (MSF) data sharing policy (Karunakara U, PLoS Med 2013). MSF is committed to sharing and disseminating health data from its programs and research in an open, timely, and transparent manner in order to promote health benefits for populations while respecting ethical and legal obligations towards patients, research participants, and their communities. The MSF data sharing policy ensures that data will be available upon request to interested researchers while addressing all security, legal, and ethical concerns. All readers may contact the generic address data.sharing@msf.org

## Abbreviations

ACEi: Angiotensin converting enzyme inhibitor
ASCVD: Atherosclerotic cardiovascular disease
CVD: Cardiovascular disease
DAZ: Dar Al Zahara
FDC: Fixed dose combination
HIV: Human Immunodeficiency Virus
LMICs: Low-and Middle-Income Countries
LSHTM: London School of Hygiene and Tropical Medicine
MoPH: Ministry of Public Health
MSF: Médecins Sans Frontières
NCD: Non-Communicable Disease
NGO: Non-Governmental Organisation
NPT: Normalisation Process Theory
UN: United Nations
WHO: World Health Organization
