# Supplementary material for: Implementation of fixed-dose combination therapy for secondary prevention of atherosclerotic cardiovascular disease among Syrian refugees in Lebanon: a qualitative evaluation
Source: BMC Health Serv Res. 2022 Jun 4;22:744. doi: 10.1186/s12913-022-08040-z (PMC9167520; doi:10.1186/s12913-022-08040-z)
Supplement: Supplementary file 1 — Additional file 1. [file 12913_2022_8040_MOESM1_ESM.docx]

## Appendix A: Topic guides 1-3 [English language version]

## Topic guide 1 for ASCVD patients receiving fixed dose combination therapy in study clinics, those who defaulted and those who opted out of treatment.

*Instructions:*

The questions do not necessarily have to be asked in the given order. Please decide which questions to ask and in what order according to the circumstances of the particular person. The idea is to allow respondents first to talk about their experience following their own logic and to feel more relaxed with the interview process. You could use prompts to lead the conversation in a particular direction, or to remind them of things they have said earlier in the conversation. Do not directly read probes to the respondent, first give them time to answer the question in their own words.

Please fill out the following details for each interview:

**Interview number:**

**Date:**

**Study ID:**

**Age:**

**Diabetes (yes or no):**

**Education:**

**Single/Married/Widow(er) (Circle one)**

**Sex:**

Sample list number:

| **Key area** | **Themes and constructs** | **Example questions** |
| --- | --- | --- |
| **Introduction** | Study aim and agencies involved  Why invited to participate  Consent & any questions |  |
| **Participant background** | Getting to know each other + building rapport | 1. Could you tell us a bit about yourself?  *Prompt:*  *When you were first diagnosed with ASCVD? How did that happen?*  *How did you first become a patient at this clinic?* |
| **Acceptability** | Intervention coherence  (The extent to which the participant understands the intervention and how it works.)  Affective attitude  (How the individual feels about the intervention.)  Burden  (The perceived amount of effort that is required to participate in the intervention.)  Self-efficacy  (The participant’s confidence that they can perform the behaviour(s) required to participate in the intervention)  Ethicality  (The extent to which the intervention has a good fit with an individual’s value system  Opportunity costs  (The extent to which benefits, profits, or values must be given up to engage in the intervention)  Focus on impact on adherence  Perceived effectiveness | 2. Since you were first diagnosed with ASCVD, can you tell me what your experience has been with treatment?  *Prompts:*  *Do you remember what kind of treatment you were prescribed in the beginning? Where was this?*  *How did you feel about it?  Did you have any challenges with that treatment?*  *Was your experience different for the different medications included in your treatment (ie were some easier to adhere to than others?)*  *Do you receive care for your heart condition in any other clinics or health facilities?*  *Who do you talk to for advice about your heart condition/treatment? (clinic staff? family? friends? pharmacist? alternative medicine practitioner, religious advisor?)*  3. Are you familiar with the new fixed-dose combination therapy treatment “Trinomia” that was introduced at this clinic? *What can you tell me about it (what does it do, what is it for)?*  *Prompts:*  *How is it different from the treatment you were on before?*  4. Do you feel that you were provided with enough information about this treatment and how it works? If not, what kind of information would have been more helpful?  *Prompts:*  *Was the information clear and in a language you could understand? Were you able to ask questions about the treatment and did you receive answers that you could understand?*  **FOR THOSE THAT OPTED INTO TRINOMIA**  I’d like to ask you now a little about your experience with the fixed dose combination therapy.  5. How do you feel overall about fixed dose therapy treatment?  *Prompts:*  *How does fixed dose combination therapy compare to your previous treatment?*  *Is there anything about it which you like better than previous treatment, or like less than previous treatment?*  6. Can you tell me about the steps involved in obtaining and taking this treatment? For example, where do you get the medicine from? How do you get it?  *Prompts:*  *Can you tell me about how you take the medicine? How does the medicince fits with your daily routine? Have you had to make any changes to your usual routine since starting Trinomia? How often do you have to take it?*  *Would you say taking this treatment is a lot of work? Why or why not?*  7. Do you feel you are easily able to incorporate the medicine into your daily life? Why or why not?  *Prompts:*  *Do you experience any challenges with the steps you described in getting or taking the medication? Can you tell me about these?*  *Are there any times when this has been easier or more difficult? Can you tell me about these times?*  *Do you experience any side effects? If yes, what were they, what did you do when you got these side effects?*  *Do you remember if you went to anyone for advice about these challenges (or side effects)? Can you tell me about this?*  *Have you sought any advice on this from anyone eg clinic, friends, pharmacy?*  8. What things are important to you when it comes to selection of a specific medicine to treat your condition? (eg. Keeps your blood pressure controlled, no side effects, low cost, easy to obtain)? Is the fixed dose combination therapy in line with these? If yes, in what way? If not, why not?  *Prompts:*  *How does the treatment fit with your treatment targets? With your lifestyle? With your daily schedule? With your budget? Do you have any specific worries about the medicine?*  *(If not mentioned above, are there any side effects that you have experienced?)*  9. What do you think has been your family’s experience of the fixed dose combination therapy?  *Prompts:*  *How does your family support your treatment?*  *Has the fixed dose combination therapy made this easier or more difficult for them in any way? How so?*  10. Is there anything that you have had to change so that you can take fixed dose combination therapy?  *Prompts:*  *Is there anything you have had to give up, stop doing or alter so that you can take fixed dose therapy treatment as prescribed?*  11. Many people find a way of taking their medicines which suits them. This might be the same or different from what the doctor has told them. Would you say that you are able to take fixed dose combination as prescribed? Has changing to this medication had any effect on your ability to take treatment as prescribed? Can you tell me about this?  *Prompts:*  *Are there any steps you take personally to make sure you take the treatment as prescribed?*  *How has the fixed dose combination therapy affected these?*  12. Overall, do you think fixed dose combination therapy is a good treatment for you? Why or why not?  **FOR THOSE WHO OPTED OUT OF TRINOMIA**  *We know that some people were offered fixed dose combination therapy but preferred not to take it. We would really like to understand how that worked, and what made people decide not to take fixed dose combination therapy and stay on their usual treatment.*  5. I understand that you opted to stay on your regular treatment rather than be switched to fixed dose combination therapy (Trinomia). Is that right? Can you tell me a little bit about why you made that choice?  *Prompts:*  *Do you remember where you got information about Trinomia from? Can you tell me about this? Do you remember if you went to anyone for advice about Trinomia? Can you tell me about this?*  *[Ask re any sources of advice mentioned by the patient earlier (Q2) - clinic, friends, pharmacy etc]*  6. If you were offered Trinomia again would you make the same choice? Why or why not?  **FOR THOSE DEFAULTED (STOPPED) ON TRINOMIA**  *We know that a few people started to take fixed dose combination therapy, and then stopped. We would really like to understand how that worked, and what made people want to stop taking fixed dose combination therapy and switch back to their previous treatment.*  5. I’d like to start by asking you a bit about your experience on fixed dose combination therapy. How did you feel overall about fixed dose therapy treatment?  *Prompts:*  *How does fixed dose combination therapy compare to your previous treatment?*  Is there anything about it which you like better than previous treatment, or like less than previous treatment?  6. And when you were taking fixed dose combination therpay, can you tell me about the steps that were involved in obtaining and taking this treatment?  *Prompts:*  *Where did you get the medicine from? How did you get it? How often did you have to take it?*  *Would you say taking this treatment was a lot of work? Why or why not?*  7. Would you say you were easily able to take all the steps you described to me above? Why or why not?  *Prompts:*  *Did you experience any challenges with the steps you described? Can you tell me about these?*  *Are there any times when this was easier or more difficult? Can you tell me about these times?*  8. What things are important to you when it comes to selection of a specific medicine to treat your condition? (eg. Keeps your blood pressure controlled, no side effects, low cost, easy to obtain)? Was the fixed dose combination therpay in line with these? If yes, in what way? If not, why not?  *Prompts:*  *How did the treatment fit with your treatment targets? With your lifestyle? With your daily schedule? With your budget?*  9. What do you think was your family’s experience of the fixed dose combination therapy?  *Prompts:*  *How does your family support your treatment?*  *Did the fixed dose combination therapy made this easier or more difficult for them in any way? How so?*  10. Is there anything that you had to change so that you could take fixed dose combination therapy?  *Prompts:*  *Is there anything you had to give up, stop doing or alter so that you can take fixed dose therapy treatment as prescribed?*  11. Many people find a way of taking their medicines which suits them. This might be the same or different from what the doctor has told them. Would you say that you were able to take fixed dose combination as prescribed? Did changing to this medication have any effect on your ability to take treatment as prescribed? Can you tell me about this?  *Prompts:*  *Are there any steps you take personally to make sure you take the treatment as prescribed?*  *Did the fixed dose combination therapy affect these?*  *Do you ever not take the medicine as prescribed? Was this because of particulary symptoms or side effects? Have you ever stopped the medicine? Why?*  12. I understand that you decided to stop taking fixed dose combination therapy and return to the treatment you were on previously. Is that right? Can you tell me a little bit about this?  *Prompts:*  *Can you tell me a bit about why you made that choice? Do you remember if you went to anyone for advice about this decision? Can you tell me about this?*  *Did you seek advice on this from anyone? [Ask re any sources of advice mentioned by the patient earlier (Q2) - clinic, friends, pharmacy etc]*  13. Have you noticed any changes since you switched back to your previous medicine?  *Prompts:*  *If there were side effects on fixed dose combination therapy, are they gone now? Do you feel better or worse than when you were on fixed dose combination therapy?*  *Is taking your treatment as prescribed easier or more difficult than when you were on fixed dose combination therapy?*  14. If you were offered Trinomia again would you choose to take it? Why or why not? |
| **Sustainability** | Challenges  Consequences  Benefits  Supportive factors | **FOR THOSE WHO OPTED INTO TRINOMIA**  1. Earlier, we discussed some of the challenges you experienced in maintaining your fixed dose combination therapy - is there anything else you could tell me about those? Is there anything that you think could reduce these challenges?  *Prompts:*  *(If do not identify any challenges could ask specifically about time, costs, information, support, drug supply, efficacy, side effects etc.)*  2. Have you experienced any negative consequences of taking (fixed dose combination therapy or regular treatment)?  *Prompts:*  *(If do not identify any challenges ask specifically about physical or psychological consequences, costs, time.)*  3. Have you experienced any positive consequences of taking (fixed dose combination therapy or regular treatment, depending on respondent)  *(If do not identify any challenges could ask specifically about physical or psychological consequences, costs, time.)*  4. *Do you think this would be a good treatment for you in the long term? Why or why not?*  *Prompts:* *What could make using (fixed dose combination therapy or regular treatment) better for you?*  *Do you feel well informed about your treatment? Could you explain why?*  *Do you feel well-supported in taking this treatment as prescribed?*  **FOR EVERYONE**  5. As I’m sure you’re aware, COVID-19 has changed the way the clinic provides services. What have been the main changes for you? How did you find that?  6. Has this affected your use of fixed dose combination therapy/ medication for your heart disease in any way? How so?  *Prompts:*  *Did the disruption have an effect on getting your medication or on how you took your medication? Did you have to switch medications because of the COVID-19 disruption? How did that make you feel?*  *Did the disruption have an effect on getting advice about your medication/condition? Did you get advice from anyone? Clinic? (nurse, doctor, phone?) [Ask re any sources of advice mentioned by the patient earlier (Q2) - friends, pharmacy etc]*  *Did you have information about who to contact or where to go if you were not able to access the clinic (for example if the clinic were closed)?*  *Do you think the disruption would have had a different effect if you were on a different treatment?*  *Is there any other information or support that you think should be offered to people taking this treatment in future?*  7. In the future, if both combination treatment (Trinomia) and separate medications were equally available, would you have a preference between the two? If so, can you explain which you would prefer and why? |
| **Thanks and**  **close** | Anything else to add  Questions  Thanks, feedback info | **FOR EVERYONE**  1. Do you have anything else to add on topic that we haven’t discussed today?  2. Do you have any questions for me?  Feedback again on how the discussion will be used and fed back. |

## Topic guide 2 for staff involved in delivering fixed dose combination therapy to ASCVD patients in study clinics

*Instructions:*

The questions do not necessarily have to be asked in the given order. Please decide which questions to ask and in what order according to the circumstances of the particular person. The idea is to allow respondents first to talk about their experience following their own logic and to feel more relaxed with the interview process. You could use probes to lead the conversation in a particular direction, or reminding them of things they have said earlier in the conversation. Do not directly read probes to the respondent, first give them time to answer the question in their own words.

| Key area | Themes | Question |
| --- | --- | --- |
| Introduction | Study aim and agencies involved  Why invited to participate  Consent & any questions? |  |
| Participant Background | Getting to know each other + building rapport | 1. Can you tell me a little about your work? What is your current role in MSF? 2. What are the responsibilities of someone in your position in the planning and implementation of health care for Syrian refugees and vulnerable Lebanese in the MSF clinic? What about for patients with cardiovascular disease specifically?   *Probes:*   - *What aspects of health care delivery are you responsible for?* - *How long have you been in your current position in this clinic? Did you hold this position elsewhere before?* |
| Acceptability | Intervention coherence  (The extent to which the participant understands the intervention and how it works.)  Affective attitude  (How the individual feels about the intervention.)  Burden  (The perceived amount of effort that is required to participate in the intervention.)  Self-efficacy  (The participant’s confidence that they can perform the behaviour(s) required to participate in the intervention)  Ethicality  (The extent to which the intervention has a good fit with an individual’s value system  Opportunity costs  (The extent to which benefits, profits, or values must be given up to engage in the intervention)  Perceived effectiveness  Focus on impact on adherence | 1. Are you aware of the fixed-dose combination medicine (“Trinomia”) that was introduced at this clinic for treatment of atherosclerotic cardiovascular disease (ASCVD) patients? What do you know about it?   *Probes:*   - *What is the goal of this medication?* - *How is it different from medication used in this clinic before?*  1. What is your overall opinion of the fixed dose combination medicine?   *Probes:*   - *How important do you think it is?* - *Do you think the introduction of fixed dose combination medicine was a positive step or a negative one? Was it both positive and negative? Why?* - *Can you explain how so? If negative, can you explain how so?*  1. What was your experience of treating patients with ASCVD before the fixed dose combination medicine was introduced?   *Probes:*   - *Did you experience any challenges, difficulties related to your job of treating patients?*  1. Has the introduction of fixed dose combination therapy changed your job experience in any way? How so?   *Probes:*   - *Has the introduction of this treatment meant changing the way you do your job in any ways? How so?* - *Does it address the challenges you experienced previously?* - *Are there new challenges?*  1. Would you say you are easily able to fulfil all the tasks required of you in relation to fixed dose combination therapy treatment? Why or why not?   *Probes:*   - *Do you feel that you have the necessary skills, time or other capacity to fulfil these tasks? Please explain.*  1. Did you have to learn any new skills in order to implement fixed dose combination therapy? Can you tell me about these?   *Probes:*   - *Did you have to learn new approaches to (chose depending on worker role and tasks previously described):* - *Prescribing medication or to monitoring treatment adherence, clinical outcomes or side effects?* - *Communicating with and informing patients?* - *Monitoring clinic activities* - *Managing the clinic team* - *Monitoring consumption of medicine*  1. Did you receive any training in these new skills? What was your impression of that?   *Probes:*   - *Do you feel the training has equipped you sufficiently to perform the tasks you are meant to perform? If not, why not?* - *Would you have preferred to have additional/further training? If so, what would this have entailed?*  1. Is there any other support provided to you to help you implement fixed dose combination therapy? Can you tell me how you feel about this support?   *Probes:*   - *Do you feel support is adequate to help you perform the tasks for which you are responsible? If not, why not?* - *Are there any other types of support you would like to see put in place to help you? If yes, what would these entail?*  1. Can you tell me a little about what you like about your job, and specifically in your role treating patients with ASCVD?   *Probes:*   - *What are the aspects that are most important to you? What aspects are most fulfilling or satisfying?*  1. How does the introduction of fixed dose combination therapy fit with the aspects of your job that you value, if at all? 2. Is there anything that you value about your job that you’ve had to give up since the introduction of fixed dose combination therapy?   *Probes:*   - *Are there any aspects of your job or tasks you used to do that you had to give up or stop doing to in order to implement this treatment protocol?*  1. Overall, would you say that fixed dose combination therapy is effective for treatment of patients? What about as a tool for staff?   *Probes:*   - *Is it a good way to achieve treatment goals? Why or why not?* - *Does it in/decrease your workload, make your job easier/more difficult?*  1. If I ask you specifically about patients’ adherence to treatment, do you think fixed dose therapy has affected patients’ adherence to prescribed treatment regimens? Why or why not?   *Probes:*   - *How does fixed dose combination therapy affect patient capacity to adhere to treatment?* - *How does the fixed dose combination therapy differ from separate treatments in terms of ease of remembering to take medication, ease of monitoring, ease of obtaining medicines, if at all?* |
| Sustainability (internal to MSF, especially MSF Lebanon) | Leadership  Capacity  Flexibility/adaptability  Interactions  Performance | I’d like to ask you a few questions now about the sustainability of the fixed dose combination medication in MSF clinics and clinics generally. We know this MSF clinic will be closing soon so when we talk about sustainability within MSF I ask you to consider MSF clinics generally, rather than this clinic specifically.   1. Do you think fixed dose combination treatment could be easily replicated in other MSF clinics in Lebanon? Why or why not?   *Probes:*   - *Do you think it would be easy to replicate in Bekaa?* - *What would be the benefits or challenges to doing so?*  1. What are the key resources that would need to be in place to support long term implementation of this treatment in MSF operations? What are the risks to these being available to support this treatment?   *Probes:*   - *Revised operational guidelines? Fixed dose combination drug procurement/supply? Health workers with appropriate skills? Continued training, capacity building, mentoring?*  1. Are there systems in place that could monitor effectiveness and sustainability of fixed dose combination for improving health outcomes among patients treated by MSF?   *Probes:*   - Are there systems for monitoring health outcomes)? - To monitor costs and benefits? - To monitor health worker and patient perspectives?  1. What is the process for you or your colleagues to provide feedback on fixed dose combination therapy for MSF patients with ASCVD?   *Probes:*   - *Do you have any examples of feedback you have given?* - *Can you share any examples of when feedback has been used to adapt the approach?*  1. If we consider the disruption to health care services caused by COVID-19, how do you think this affected implementation of the fixed dose combination medication?   *Probes:*   - Did this affect the drug supply chain? Patient ability to obtain medicines? Health worker opportunities to prescribe, inform patients about or monitor this treatment? How so?  1. Was anything done to respond to these disruptions? Can you describe these responses? 2. What could be done in the future to prevent any negative impacts that health service disruptions like COVID-19 had on delivery of fixed dose combination treatment to patients with ASCVD?   *Probes:*   - Could changes be made to how and from where drugs are procured? - To where drugs are dispensed (for eg in the community vs at clinics)? - To the number of pills patients are prescribed per visit (meaning how long the supply will last them)? - How treatment is monitored? How consultations with patients are organised? - How so?  1. Do you think treatment using fixed dose combination therapy was in any way more or less resilient to the disruptions caused by COVID-19? How so?   *Probes:*   - *Are there differences in how often patient need to obtain medicines, be followed up, etc?*   FOR SENIOR MANAGEMENT STAFF ONLY   1. How do you see the role of someone in your position in supporting the delivery of treatment with fixed dose combination therapy over the long term in MSF clinics in Lebanon?   *Probes:*   - *How confident do you feel about the capacity of someone in your role to support long-term implementation of fixed dose combination therapy treatment? Why or why not?* - *What changes would have to be made to your role to ensure sustained delivery of this medication?*   Finally, I would like to ask your thoughts about the sustainability of fixed dose combination therapy in MSF contexts beyond Lebanon.   1. How easy do you think would it be to implement fixed dose combination treatment in other humanitarian crisis settings where MSF works?   *Probes:*   - *What would need to be in place for this approach to ‘work’ in other MSF settings?* - *Is there something in particular that you would recommend to decision-makers in other MSF settings if they wanted to use this or a similar approach?*  1. What could be done to improve the potential sustainability of the treatment approach in MSF clinics outside of Lebanon?   *Probes:*   - Could changes be made to overall operational guidelines? - Changes to health worker training? - Changes to drug procurement? - Changes to engagement with actors outside of MSF? - Other organisational or policy changes? - How so? |
| Sustainability (external to MSF) |  | FOR SENIOR MANAGEMENT STAFF ONLY  **I would like to ask you now a few questions to understand your views on the sustainability of a treatment like fixed dose combination therapy in Lebanon beyond MSF operations**   1. Beyond MSF, what organisations would be key in supporting further implementation of this treatment protocol for vulnerable populations in Lebanon? What roles might they play?   *Probes:*   - *MOPH, MOSA, YMCA, ICRC, WHO….?*  1. Do you have an impression of how these organisations work together? How do they work with MSF?   *Probes:*   - *How are responsibilities for delivering health care shared among them?* - *How are decisions made about what kind of interventions to prioritise?*  1. If MSF were to support the implementation of a treatment like fixed dose combination therapy to vulnerable populations in Lebanon outside of MSF clinics, what specific support do you think MSF could provide?   *Probes:*   - *What organisations could they lend support to?* - *What specific tasks or activities could MSF be responsible for? What about someone in your role?*  1. How confident do you feel in MSF’s capacity to fulfil this kind of role in supporting implementation of this treatment ? Why or why not?   *Probes:*   - *Does MSF currently fulfil this type of role in other aspects of health services? Does it experience any challenges in doing so?* - *Do you foresee any challenges related to fulfilling this role in relation to fixed dose combination therapy specifically?*  1. What changes would MSF have to make to ensure sustained delivery of this treatment approach?   *Probes:*   - *Are there ways in which MSF would have to change activities to address some of the challenges you mentioned above?*  1. What benefits would MSF see in wider implementation of fixed dose combination therapy to vulnerable populations in Lebanon?   *Probes:*   - *Eg. Better health outcomes for patients as key MSF mission; alignment across agencies and health care providers supports treatment continuity*  1. And what do you think could be the costs to MSF, if any, if MSF were to support wider implementation of fixed dose combination therapy?   *Probes:*   - *Eg. Would MSF be called on to support staff training?*  1. How confident do you feel the capacity of other key organisations to be able to fulfil their potential role in supporting continued implementation of this treatment? Why or why not?   *Probes:*   - *In your view, do these organisations currently experience any challenges in fulfilling this type of role?* - *Do you foresee any challenges they might face related to fulfilling their role in relation to fixed dose combination therapy?*  1. What are the key resources or systems that need to be in place to support long term implementation of this treatment in Lebanon for refugees and vulnerable Lebanese? What are the risks to these being available to support this treatment protocol?   *Probes:*   - *Are there specific financial, human resource, clinical, legislative or other resources that are required?*  1. Overall, what do you think would be the main challenges to the sustainability of treatment with fixed dose combination therapy beyond MSF clinics in Lebanon?   *Probes:*   - *Would there be challenges with health worker or patient knowledge? Policy maker attitudes? Drug supply? Availability or affordability of the drug?*  1. Overall, what factors do you think support the sustainability of treatment with fixed dose combination therapy beyond MSF clinics in Lebanon?   *Probes:*   - *Acceptability of the treatment to patients? Lower cost?*  1. What could be done to improve the sustainability of the treatment approach in Lebanon in general?   *Probes:*   - *Are changes required to financing of health care for vulnerable populations? To provision of care for vulnerable populations? To national production, procurement or supply of medicines? To clinical guidelines? To medical training curricula? To health worker or patient knowledge, attitudes and practices?*  1. Are there structures in place that could be used to monitor the effectiveness of fixed dose combination for improving health outcomes among vulnerable populations with ASCVD in Lebanon?   *Probes:*   - *Hospital or clinic registries? Monitoring of acute cardiovascular event incidence?* |
| Thanks and  close | Anything else to add  Questions  Thanks, feedback info | Is there anything else on the topic of fixed dose combination therapy that we haven’t discussed that you would like to add?  Do you have any questions for me?  Provide feedback again on how the discussion will be used and how findings will be shared. |

**Topic guide 3 for stakeholders relevant to fixed dose combination therapy for ASCVD patients**

*Instructions:*

The questions do not necessarily have to be asked in the given order. Please decide which questions to ask and in what order according to the circumstances of the particular person. The idea is to allow respondents first to talk about their experience following their own logic and to feel more relaxed with the interview process. You could use probes to lead the conversation in a particular direction, or remind them of things they have said earlier in the conversation. Do not directly read probes to the respondent, first give them time to answer the question in their own words.

| Key area | Themes | Question |
| --- | --- | --- |
| Introduction | Study aim and agencies involved  Why invited to participate  Consent & any questions? |  |
| Participant Background | Getting to know each other + building rapport  Establishing participant perspective | 1. Can you tell me a little about your work? What is your role in (organisation/department)? 2. What is the role of someone in your position in the planning and implementation of health care for Syrian refugees in Lebanon? What about for ASCVD specifically?   *Probes:*   - *What aspects of health care delivery are you responsible for?* - *How long have you been in this position?* |
| Acceptability |  | 1. Are you aware of the MSF treatment protocol to use fixed dose combination therapy (FDC) for people who have had a stroke or heart disease in Abdeh/Dar Al Zahara clinics? What do you know about it?   ***If they are not aware, explain the protocol briefly***  *Probes:*   - *What is the goal of this treatment protocol? How is it different from the treatment protocol used in this clinic before?*  1. What is your overall opinion of the fixed dose therapy treatment protocol?   ***If they are hearing about it for the first time: “What are your initial thoughts about the treatment protocol I have just described to you?”***  *Probes:*   - *How important do you think this treatment protocol is?* - *What are the advantages of fixed dose combination therapy?* - *What are the disadvantages?*  1. Have you or your organisation been involved in this treatment protocol? In what ways?     *Probe:*   - *Have you had input into the development of the protocol? How so?* - *Have you had input into the implementation of the protocol? How so?*  1. Can you tell me a little about the goals or values of your organisation in terms of health care for Syrian refugees?   *Probes:*   - Are there any formal commitments your organisation has with regard to health care for Syrian refugees, for example to ensure equity in care, affordable care, high quality care, etc?  1. Do you think the treatment protocol is in line with or supports achievement of your organisation’s goals and values? How so?   *Probes:*   - Does this treatment protocol affect access to treatment in positive ways, for eg ensuring everyone is able to access the treatment they need when they need it, ensuring treatment is affordable, etc? - Does it meet the needs of the most vulnerable patients? - If not, how could it better do so? |
| Sustainability | Interactions  Leadership  Capacity  Flexibility/Adaptability  Performance | **I would like to ask you now a few questions to understand your views on the sustainability of a treatment protocol like fixed dose combination therapy beyond MSF programmes.**   1. Beyond MSF, what organisations would be key in ensuring continued implementation of this treatment protocol for vulnerable populations in Lebanon? What are their roles?   *Probes:*   - *MOPH, MOSA, YMCA, ICRC, WHO….?*  1. How do these organisations work together?   *Probes:*   - *How are responsibilities for delivering health care shared among them?* - *How are decisions made about what kind of interventions to prioritise?*      1. How would you see the role of someone in your position or that of your organisation in supporting the continued implementation of a treatment like fixed dose combination therapy to the wider population?     *Probes:*   - *What specific tasks or activities would your organisation be responsible for? What about someone in your role?*  1. How confident do you feel in in your organisation’s capacity to fulfil this role in supporting continued implementation of this treatment protocol? Why or why not?     *Probes:*   - *Do you currently experience any challenges in fulfilling this type of role?* - *Do you foresee any challenges related to fulfilling this role in relation to fixed dose combination therapy?*  1. What changes would your organisation have to make to ensure sustained delivery of this treatment approach?   *Probes:*   - *Are there ways in which you would have to change activities to address some of the challenges you mentioned above?*  1. *What would the benefits to your organisation be of fixed dose combination therapy?*   *Probes:*   - *Eg. Better patient adherence, fewer costly acute health care events, fewer drugs to procure*  1. *What would the costs to your organisation be of fixed dose combination therapy?*   *Probes:*   - *Eg. Requires re-training staff, more complicated drug procurement*  1. How confident do you feel the capacity of other key organisations to fulfil their role in supporting continued implementation of this treatment protocol? Why or why not?   *Probes:*   - *In your view, do these organisations currently experience any challenges in fulfilling this type of role?* - *Do you foresee any challenges they might face related to fulfilling their role in relation to fixed dose combination therapy?*  1. What are the key resources that need to be in place to support long term implementation of this treatment protocol? What are the risks to these being available to support this treatment protocol?   *Probes:*   - *Are there specific financial, human resource, clinical, political or other resources that are required?* - *Are there any guidelines or other supporting resources that are required?*  1. If we consider disruption to health care services like that caused by COVID-19, how do you think this would affect sustainability of the fixed dose combination therapy treatment protocol?   *Probes:*   - *What effect does it have on access to care, supply of drugs, human resources…?*  1. What could be done to prevent any negative impacts that health service disruptions like this have on delivery of treatment including fixed dose combination therapy to patients?   *Probes:*   - *Eg. Requires re-training staff, more complicated drug procurement*  1. Do you think treatment using fixed dose combination therapy would be in any way more or less resilient to the disruptions caused by COVID-19? How so?   *Probes:*   - *Eg. More or less difficult to procure, requires fewer clinic visits, more difficult to monitor*  1. How easy do you think would it be to implement this treatment protocol in other humanitarian crisis settings?   *Probes:*   - *What would need to be in place for this approach to ‘work’ in other settings?* - *Is there something in particular that you would recommend to decision-makers in other settings if they wanted to use this or a similar approach?*  1. Overall, what do you think would be the main challenges to the sustainability of treatment with fixed dose combination therapy beyond MSF clinics?   *Probes:*   - *Would there be challenges with health worker or patient knowledge? Policy maker attitudes? Drug supply?*  1. Overall, what factors do you think support the sustainability of treatment with fixed dose combination therapy beyond MSF clinics?   *Probes:*   - *Acceptability of the treatment to patients? Lower cost?*  1. What could be done to improve the sustainability of the treatment approach beyond MSF clinics? 2. Are there structures in place that could be used to monitor the continued effectiveness of this treatment approach for improving health outcomes among Syrian refugees with ASCVD? |
| Thanks and  close | Anything else to add  Questions  Thanks, feedback info | 1. Is there anything else you would like to say about your views on the fixed dose combination therapy approach, its sustainability, or about treatment of Syrian refugees with ASCVD generally? 2. Do you have any questions for me?   Feedback again on how the discussion will be used and fed back. |
