# Supplementary material for: Implementation of fixed-dose combination therapy for secondary prevention of atherosclerotic cardiovascular disease among Syrian refugees in Lebanon: a qualitative evaluation
Source: BMC Health Serv Res. 2022 Jun 4;22:744. doi: 10.1186/s12913-022-08040-z (PMC9167520; doi:10.1186/s12913-022-08040-z)
Supplement: Supplementary file 2 — Additional file 2. [file 12913_2022_8040_MOESM2_ESM.docx]

Appendix B: Recruitment approach, data collection mode and setting for each participant group.

| **Participant Group** | **Method of approach** | **Setting and mode of data collection** | **Presence of non-participants** |
| --- | --- | --- | --- |
| Patients | Initial contact by telephone prior to next scheduled clinic visit at clinic.  Face-to-face follow up at clinic visit. | Conducted face-to-face in private room at clinic, scheduled to coincide with routine clinic visit. | None |
| Staff | Face-to face, telephone or email, dependent on staff role and presence in clinic. | Conducted face-to-face in private room at clinic, or remotely using Zoom software. | None |
| External Stakeholders | Email | Conducted remotely using Zoom software. | None |
